# PrEP initiation and discontinuation among transgender women in the United States: a longitudinal, mixed methods cohort study

**DOI:** 10.1002/jia2.26199

**Published:** 2023-12-20

**Authors:** Erin E. Cooney, Haneefa T. Saleem, Meg Stevenson, Rodrigo A. Aguayo‐Romero, Keri N. Althoff, Tonia C. Poteat, S. Wilson Beckham, Dee Adams, Asa E. Radix, Andrew J. Wawrzyniak, Christopher M. Cannon, Jason S. Schneider, J. Sonya Haw, Allan E. Rodriguez, Kenneth H. Mayer, Chris Beyrer, Sari L. Reisner, Andrea L. Wirtz, American Cohort to Study HIV Acquisition Among Transgender Women (LITE) Study Group

**Affiliations:** ^1^ Department of International Health Johns Hopkins Bloomberg School of Public Health Baltimore Maryland USA; ^2^ Department of Epidemiology Johns Hopkins Bloomberg School of Public Health Baltimore Maryland USA; ^3^ Division of Endocrinology, Diabetes, and Hypertension Brigham and Women's Hospital Boston Massachusetts USA; ^4^ Department of Medicine Harvard Medical School Boston Massachusetts USA; ^5^ Fenway Health The Fenway Institute Boston Massachusetts USA; ^6^ Department of Social Medicine University of North Carolina Chapel Hill North Carolina USA; ^7^ Department of Health Behavior and Society Johns Hopkins Bloomberg School of Public Health Baltimore Maryland USA; ^8^ Callen‐Lorde Community Health Center New York New York USA; ^9^ Department of Psychiatry and Behavioral Sciences University of Miami Miller School of Medicine Miami Florida USA; ^10^ Whitman‐Walker Institute Washington DC USA; ^11^ Department of Medicine Emory University School of Medicine Atlanta Georgia USA; ^12^ Division of Endocrinology, Metabolism and Lipids Emory University School of Medicine Atlanta Georgia USA; ^13^ Division of Infectious Diseases Department of Medicine University of Miami Miller School of Medicine Miami Florida USA; ^14^ Duke University Global Health Institute Durham North Carolina USA; ^15^ Department of Epidemiology Harvard TH Chan School of Public Health Boston Massachusetts USA

**Keywords:** pre‐exposure prophylaxis, transgender women, prevention‐effective adherence, PrEP discontinuation, PrEP initiation, discontinuation typologies

## Abstract

**Introduction:**

Transgender women in the United States experience high HIV incidence and suboptimal Pre‐exposure prophylaxis (PrEP) engagement. We sought to estimate PrEP initiation and discontinuation rates and characterize PrEP discontinuation experiences among a prospective cohort of transgender women.

**Methods:**

Using a sequential, explanatory, mixed‐methods design, 1312 transgender women at risk for HIV acquisition were enrolled from March 2018 to August 2020 and followed through July 2022 (median follow‐up 24 months; interquartile range 15–36). Cox regression models assessed predictors of initiation and discontinuation. In‐depth interviews were conducted among 18 participants, including life history calendars to explore key events and experiences surrounding discontinuations. Qualitative and quantitative data were integrated to generate typologies of discontinuation, inform meta‐inferences and facilitate the interpretation of findings.

**Results:**

21.8% (*n* = 286) of participants reported taking PrEP at one or more study visits while under observation. We observed 139 PrEP initiations over 2127 person‐years (6.5 initiations/100 person‐years, 95% CI: 5.5–7.7). Predictors of initiation included identifying as Black and PrEP indication. The rate of initiation among those who were PrEP‐indicated was 9.6 initiations/100 person‐years (132/1372 person‐years; 95% CI: 8.1–11.4). We observed 138 PrEP discontinuations over 368 person‐years (37.5 discontinuations/100 person‐years, 95% CI: 31.7–44.3). Predictors of discontinuation included high school education or less and initiating PrEP for the first time while under observation. Four discontinuation typologies emerged: (1) seroconversion following discontinuation; (2) ongoing HIV acquisition risk following discontinuation; (3) reassessment of HIV/STI prevention strategy following discontinuation; and (4) dynamic PrEP use coinciding with changes in HIV acquisition risk.

**Conclusions:**

PrEP initiation rates were low and discontinuation rates were high. Complex motivations to stop using PrEP did not consistently correspond with HIV acquisition risk reduction. Evidence‐based interventions to increase PrEP persistence among transgender women with ongoing acquisition risk and provide HIV prevention support for those who discontinue PrEP are necessary to reduce HIV incidence in this population.

## 1 INTRODUCTION

Transgender women experience a high and disproportionate burden of HIV [1, 2]. Many socio‐structural drivers of high HIV incidence among transgender women are also barriers to engagement in the HIV prevention cascade [3, 4, 5, 6]. Observational data on longitudinal PrEP engagement among transgender women are limited [7, 8], but data from clinical cohorts suggest that PrEP persistence among transgender women is low [9, 10]. A daily, oral PrEP demonstration study with multiple sites in Latin America conducted from 2018 to 2021, ImPrEP, found less than half of transgender women remained on PrEP 52 weeks after initiation [11].

As Haberer and colleagues have described, PrEP is effective so long as adherence is high during periods of increased HIV acquisition risk that are not covered by other effective prevention strategies (e.g. condoms)—a paradigm coined “prevention‐effective adherence” [12]. Therefore, as both PrEP use and clinician understanding of prevention‐effective adherence practices become more widespread, longitudinal aggregate measures of PrEP engagement (e.g. PrEP persistence at 1 year) should be analysed with consideration for dynamic (i.e. time‐varying as opposed to fixed) HIV acquisition risk [13].

Prevention‐effective adherence has the potential to maximize PrEP efficiency and increase feasibility and acceptability for potential users [14]. However, real‐world evidence suggests that not all individuals are able to selectively use PrEP during periods of increased HIV acquisition risk; seroconversions among former PrEP users and high HIV incidence during gaps in PrEP use show that this strategy may not successfully meet the HIV prevention needs of all [15]. Velloza and colleagues found that adolescent girls and young women in HIV Prevention Trials Network 082 successfully engaged in prevention‐effective adherence practices [16], while our own work with transgender women demonstrated just 28% engaged in prevention‐effective adherence practices [17]. Thus, there is a need to better characterize PrEP discontinuation events so that discontinuations that coincide with reductions in HIV acquisition risk can effectively be disaggregated from discontinuations that occur in the context of ongoing HIV acquisition risk.

Given the paucity of data on PrEP outcomes among transgender women in the United States, we aimed to describe PrEP initiation and discontinuation rates in a large, multi‐site, observational cohort study comprised exclusively of transgender women in the United States. To further understand the extent to which PrEP discontinuations were consistent with prevention‐effective adherence practices, we aimed to develop PrEP discontinuation typologies. Through the identification of discontinuation typologies, we endeavour to inform interventions designed to meet the HIV prevention needs of transgender women who discontinue PrEP and remain at risk of HIV acquisition.

## 2 METHODS

### 2.1 Study design and participants

The LITE Study was a multi‐site, epidemiologic cohort focused on HIV prevention, including PrEP engagement, among transgender women in the United States. English‐ and/or Spanish‐speaking adult transgender women residing in the eastern and southern United States were eligible. Cohort enrolment was restricted to transgender women who were confirmed to not be living with HIV. The LITE Study is unique in its inclusion of a site‐based, technology‐enhanced mode (consisting of annual in‐person socio‐behavioural surveys and HIV testing with remote surveys and HIV tests completed quarterly) as well as an exclusively digital mode (surveys and HIV tests completed exclusively remotely). Study protocols for each mode have been published previously [18, 19]. Survey domains included socio‐demographics, gender‐affirming experiences, general physical and mental health, substance use, sexual health, experiences of discrimination and violence, healthcare engagement and PrEP indication and engagement (Panel S1). HIV/STI testing, safer sex counselling and referrals to local treatment and prevention services, including PrEP (as needed), were provided by trained study staff. Protocol development and study implementation were guided by a Community Advisory Board and formative, qualitative research [20, 21, 22]. Study procedures were approved by the Johns Hopkins School of Medicine Institutional Review Board. Written informed consent was obtained from all participants before enrolment.

### 2.2 Procedures

We enrolled participants from March 2018 until August 2020. At enrolment, participants consented to participate for 24 months. Those who completed 24 months of follow‐up between March 2020 and January 2022 were invited to continue participating for an additional 6–24 months (up to a maximum of 48 months with final study visits occurring on or before 30th July 2022). Participants generally completed surveys semi‐annually, with site‐based participants completing quarterly surveys for the first 24 months of enrolment.

Qualitative data were collected between February and June 2022 as part of a sub‐study designed to explore multilevel influences on PrEP engagement among transgender women [23]. In‐depth interviews were conducted with a subset of 18 LITE participants. Two interviewers with extensive training in qualitative research methods, who had built rapport with LITE participants over several years as participant‐facing LITE study staff, consented participants and conducted the interviews over Zoom in English and/or Spanish, according to participant preference. We used life‐history methods to structure the discussion, which included sections on key events across the lifespan, healthcare engagement and PrEP experiences [24, 25]. All interviews were audio‐recorded, transcribed, checked for accuracy by the interviewer and coded in the interview language. Key quotes were translated from Spanish to English by the team member who conducted the interviews.

### 2.3 Statistical analysis

We ascertained PrEP experience at enrolment by asking participants to self‐report history of PrEP use and current PrEP use (Panel S1). To calculate PrEP initiation rates, we divided the number of observed PrEP initiations (events) by the number of person‐years contributed. Those who were not current PrEP users at baseline (i.e. both former PrEP users and those who were PrEP‐naive) contributed person‐time from enrolment until censoring. We censored data at the first reported PrEP use for PrEP initiators, the last visit in which PrEP use data were available for non‐initiators or time of seroconversion for those who seroconverted and never initiated PrEP. To calculate PrEP discontinuation rates, we divided the number of PrEP discontinuation events by the number of person‐years observed while on PrEP. Those who reported current PrEP use at baseline contributed person‐time from enrolment until censoring. Observations were censored at last reported PrEP use for those who discontinued or the last visit in which PrEP use data were available for those who did not discontinue. For those who initiated PrEP while under observation, person‐time was contributed from time of initiation until censoring. We used a continuous time‐to‐event approach and Cox regression models to estimate the relative risks of PrEP initiation and discontinuation by socio‐demographics and PrEP experience at enrolment. Statistically significant (*p*<0.05) predictors in bivariate analyses were retained in multivariable models, along with age, race, ethnicity and census region. All quantitative analyses were conducted using Stata 17.

### 2.4 Qualitative analysis and data integration

As qualitative data collection progressed, weekly analytic meetings among study team members were held. As our understanding of the factors and events that shaped PrEP discontinuations improved, we iteratively refined the emerging discontinuation typologies and implemented theoretical sampling to incorporate data from participants with experiences that would complement the data already collected. Sampling continued until code and meaning saturation were achieved. In‐depth interview transcripts were imported into Atlas.ti for analysis. Deductive codes were derived from the interview guide, while inductive codes were generated based on analytic memos, close reading of transcripts and line‐by‐line coding.

Vertical and horizontal analysis in mixed methods research refers to the analysis of data within an individual participant and across multiple participants, respectively. Vertical analysis integrated qualitative and quantitative data (interview transcripts, survey responses, STI/HIV testing and treatment records) for each participant to generate PrEP engagement narratives, which established the sequence and timing of key life events and experiences relative to PrEP discontinuation. Horizontal analysis of these PrEP engagement narratives then led to the identification of cross‐cutting and salient themes.

Data were integrated by comparing qualitative and quantitative findings and interpreting the results of each with consideration for the extent to which findings converged or diverged [26]. Qualitative findings provided context and depth to quantitative findings. Throughout the analysis, typologies of discontinuation emerged and were refined. Data integration culminated with a joint display characterizing typologies of PrEP discontinuation.

### 2.5 Role of the funding source

The funders of this study had no role in the collection, analysis or interpretation of data; in the writing of the report; nor the decision to submit the paper for publication.

## 3 RESULTS

### 3.1 Participant characteristics

From March 2018 to August 2020, we enrolled 1312 transgender women (Figure S1). Socio‐demographic characteristics are presented in Table 1. Approximately half experienced PrEP indication at enrolment and 69.0% were PrEP‐indicated on at least one occasion over follow‐up (Table 1). While under observation, 21.8% of participants (*n* = 286) reported taking PrEP at one or more study visits, including 35 former PrEP users at enrolment (12.2%), 147 current PrEP users at enrolment (51.4%) and 102 who initiated PrEP for the first time after enrolment (35.7%).

**Table 1:** Participant socio‐demographics and PrEP engagement characteristics by PrEP discontinuation status among transgender women enrolled in the LITE cohort and under observation March 2018–July 2022 (*n* = 1302^a^)

		Those who reported PrEP use while under observation		Those who did not report PrEP use while under observation	
Factor	Total	Did not discontinue PrEP	Discontinued PrEP	Never initiated PrEP	*p*‐value
*N*	1302	148	138	1016	
Age, median (IQR)	28 (23, 35)	29 (25, 35)	29 (24, 38)	28 (23, 35)	0.045
Age category					<0.001
Youth (18–24 years)	423 (32.5%)	29 (19.6%)	41 (29.7%)	353 (34.7%)	
Age 25+ years	879 (67.5%)	119 (80.4%)	97 (70.3%)	663 (65.3%)	
Ethnoracial identity					<0.001
Non‐Hispanic White	697 (53.5%)	45 (30.4%)	48 (34.8%)	604 (59.4%)	
Non‐Hispanic Black	170 (13.1%)	34 (23.0%)	34 (24.6%)	102 (10.0%)	
Hispanic White	86 (6.6%)	9 (6.1%)	14 (10.1%)	63 (6.2%)	
Hispanic Black	22 (1.7%)	4 (2.7%)	3 (2.2%)	15 (1.5%)	
Non‐Hispanic and more than one race or other race	181 (13.9%)	21 (14.2%)	17 (12.3%)	143 (14.1%)	
Hispanic and more than one race or other race	130 (10.0%)	33 (22.3%)	20 (14.5%)	77 (7.6%)	
Unknown	16 (1.2%)	2 (1.4%)	2 (1.4%)	12 (1.2%)	
Black racial identity (inclusive of those who identify as Black and another racial or ethnic identity)					<0.001
No	1012 (78.4%)	89 (61.0%)	85 (62.5%)	838 (83.1%)	
Yes	279 (21.6%)	57 (39.0%)	51 (37.5%)	171 (16.9%)	
Hispanic/Latina (inclusive of all racial identities)					<0.001
No	1059 (81.6%)	102 (68.9%)	101 (73.2%)	856 (84.7%)	
Yes	238 (18.4%)	46 (31.1%)	37 (26.8%)	155 (15.3%)	
Census region					0.013
Northeast	565 (43.4%)	61 (41.2%)	60 (43.5%)	444 (43.7%)	
Midwest	146 (11.2%)	10 (6.8%)	7 (5.1%)	129 (12.7%)	
South	591 (45.4%)	77 (52.0%)	71 (51.4%)	443 (43.6%)	
Cohort mode					<0.001
Site‐based technology enhanced	715 (54.9%)	118 (79.7%)	106 (76.8%)	491 (48.3%)	
Exclusively digital	587 (45.1%)	30 (20.3%)	32 (23.2%)	525 (51.7%)	
Year of study enrolment					<0.001
2018	417 (32.0%)	78 (52.7%)	68 (49.3%)	271 (26.7%)	
2019	812 (62.4%)	63 (42.6%)	67 (48.6%)	682 (67.1%)	
2020	73 (5.6%)	7 (4.7%)	3 (2.2%)	63 (6.2%)	
Income					0.003
Above the federal poverty level	698 (53.6%)	76 (51.4%)	60 (43.5%)	562 (55.3%)	
Below the federal poverty level	410 (31.5%)	55 (37.2%)	61 (44.2%)	294 (28.9%)	
Unknown	194 (14.9%)	17 (11.5%)	17 (12.3%)	160 (15.7%)	
Educational background					<0.001
High school diploma/GED or less	357 (27.4%)	45 (30.4%)	62 (44.9%)	250 (24.6%)	
Some college or higher	934 (71.7%)	101 (68.2%)	74 (53.6%)	759 (74.7%)	
Unknown	11 (0.8%)	2 (1.4%)	2 (1.4%)	7 (0.7%)	
Health insurance^b^					<0.001
Uninsured	213 (16.9%)	22 (15.5%)	22 (16.5%)	169 (17.1%)	
Public insurance (e.g. Medicaid or Medicare)	543 (43.0%)	76 (53.5%)	76 (57.1%)	391 (39.6%)	
Private insurance	507 (40.1%)	44 (31.0%)	35 (26.3%)	428 (43.3%)	
Experienced homelessness^b^					0.001
No	1005 (77.4%)	108 (73.0%)	92 (66.7%)	805 (79.5%)	
Yes	293 (22.6%)	40 (27.0%)	46 (33.3%)	207 (20.5%)	
PrEP indicated at enrolment					<0.001
No	688 (52.9%)	40 (27.0%)	38 (27.5%)	610 (60.2%)	
Yes	612 (47.1%)	108 (73.0%)	100 (72.5%)	404 (39.8%)	
PrEP indicated over follow‐up					<0.001
No	403 (31.0%)	6 (4.1%)	8 (5.8%)	389 (38.3%)	
Yes	899 (69.0%)	142 (95.9%)	130 (94.2%)	627 (61.7%)	
PrEP experience at enrolment					<0.001
PrEP naive	1054 (81.1%)	47 (32.2%)	55 (39.9%)	952 (93.7%)	
Former PrEP user	99 (7.6%)	17 (11.6%)	18 (13.0%)	64 (6.3%)	
Current PrEP user	147 (11.3%)	82 (56.2%)	65 (47.1%)	0 (0.0%)	
Year of PrEP initiation (among those who initiated)					0.003
2018	16 (11.5%)	8 (12%)	8 (11%)		
2019	59 (42.4%)	25 (38%)	34 (47%)		
2020	39 (28.1%)	13 (20%)	26 (36%)		
2021	18 (12.9%)	13 (20%)	5 (7%)		
2022	7 (5.0%)	7 (11%)	0 (0%)		

^a^ There were *n* = 1312 cohort participants; *n* = 10 declined to respond to questions on current and/or previous PrEP use at enrolment and are, therefore, excluded from the analytic sample. Participants who declined to respond to questions on socio‐demographic characteristics are coded as “unknown” in Table 1. ^b^Participants were considered uninsured and to have experienced homelessness if uninsured status and recent homelessness were reported at any point over follow‐up, respectively. The remaining characteristics were reported based on response at enrolment (except where noted).

Participants who never engaged in PrEP use while under observation predominately identified as non‐Hispanic White (Table 1). The majority were college‐educated and experienced PrEP‐indication over follow‐up. Those who used PrEP under observation and did not discontinue as of last follow‐up were disproportionately comprised of those aged 25 and older, were more likely to identify as Black and/or Hispanic/Latina and the majority were using PrEP at enrolment. Among those who discontinued PrEP while under observation, a disproportionate number had income below the federal poverty level, were publicly insured and experienced homelessness over follow‐up.

Eighteen in‐depth interviews were conducted with transgender women ranging in age from 18 to 50 years old. Participants had diverse ethnoracial identities (Table S1). A majority resided in the South and participated in site‐based mode. All participants experienced PrEP indication over follow‐up. Additional socio‐demographic characteristics of the qualitative subsample are reported in Table S1.

### 3.2 Incidence of PrEP initiation and discontinuation

**Figure 1:**
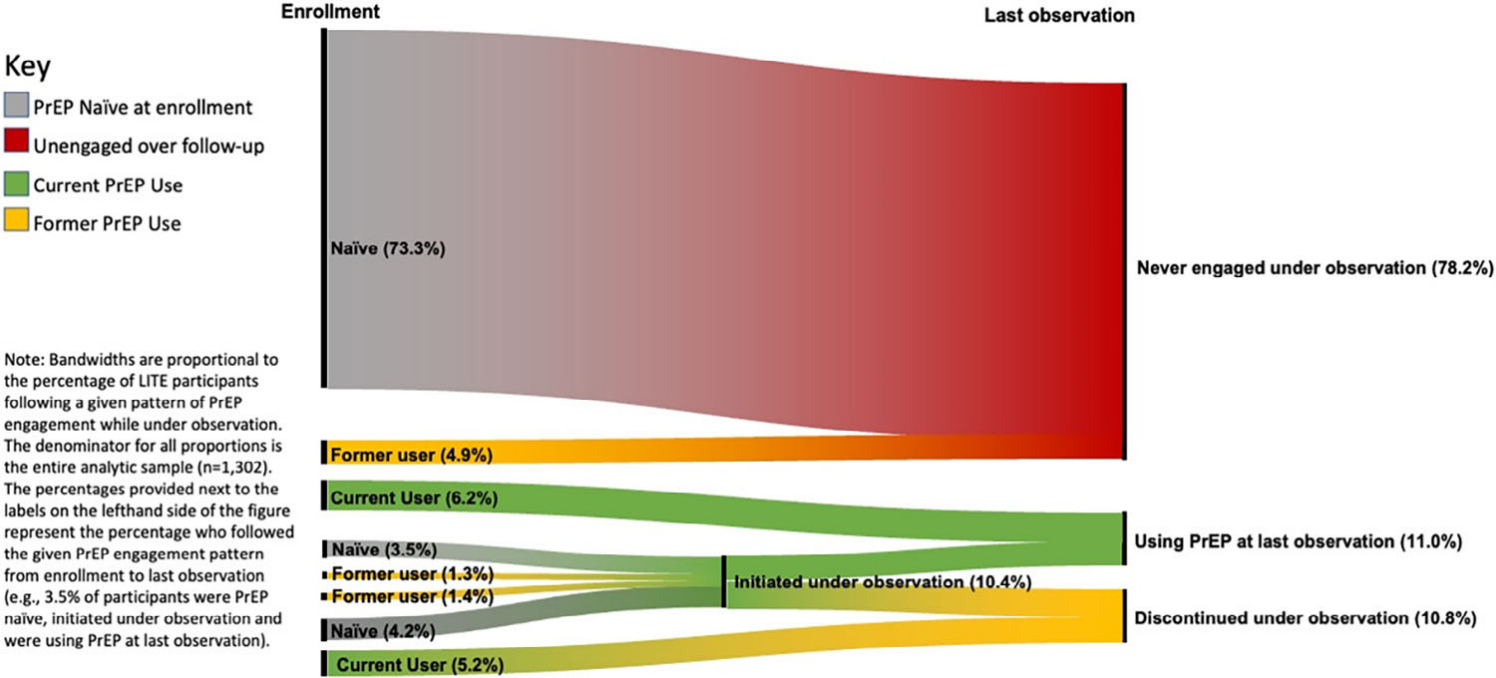
PrEP initiations and discontinuations among transgender women in the United States enrolled in The LITE Cohort under observation from March 2018 to July 2022 (*n* = 1302).

We observed 139 PrEP initiations over 2127 person‐years (6.5 initiations/100 person‐years, 95% CI: 5.5–7.7). We observed 102 initiations among those who were PrEP naive and 35 re‐initiations among those who were former PrEP users at enrolment. The rate of initiation among those who were PrEP‐indicated was 9.6 initiations/100 person‐years (132/1372 person‐years; 95% CI: 8.1–11.4). We observed 138 discontinuations over follow‐up including 55 among those who had initiated PrEP for the first time while under observation and 83 among those whose first initiation occurred prior to enrolment. The rate of PrEP discontinuation was 37.5 discontinuations/100 person‐years over (138/368 person‐years, 95% CI: 31.7–44.3; Figure 1).

Three participants reported initiation of injectable PrEP, two of whom had previously discontinued oral PrEP (24 and 36 months prior) and one of whom was an adherent user of daily, oral PrEP for 2 years prior to switching. Among the 286 cohort participants who engaged with PrEP over follow‐up, 18.9% (*n* = 54) reinitiated PrEP at least once after discontinuation.

Incidence rates of PrEP initiation and discontinuation are reported by socio‐demographic characteristics and former PrEP experience in Table 2. Those who identified as Black had PrEP initiation rates that were more than three times higher than those who did not identify as Black, although rates of discontinuation did not differ by race. Similarly, those who identified as Hispanic/Latina initiated PrEP at a rate 73% greater than those who did not identify as Hispanic/Latina, with no difference in discontinuation rates. Those residing in the Northeast and South had higher initiation rates than those in the Midwest, while all regions had similar rates of discontinuation.

**Table 2:** Rates of PrEP initiation and discontinuation by socio‐demographics and PrEP experience among transgender women in the United States enrolled in The LITE Cohort and under observation March 2018–July 2022

	PrEP initiations (among *n*=1155 not currently using PrEP at enrolment)							PrEP discontinuations (among *n*=286 PrEP users; inclusive of 147 PrEP users at enrolment and 139 who initiated PrEP over follow‐up)						
	% of participants who initiated	*p*‐value (Pearson chi^2^)	# of events	Person‐years (pys)	Rate (per 100 pys)	*p*‐value (log‐rank test)	95% confidence interval	% of participants who discontinued	*p*‐value (Pearson chi^2^)	# of events	Person‐years (pys)	Rate (per 100 pys)	*p*‐value (log‐rank test)	95% confidence interval
Overall	12.0% (139/1155)	–	139	2126.8	6.5	–	(5.5, 7.7)	48.3% (138/286)	–	138	368	37.5	–	(31.7, 44.3)
Age														
18—24	9.7% (38/391)	0.084	38	666.8	5.7	0.2082	(4.1, 7.8)	58.6% (41/70)	0.047	41	68.6	59.6	0.0093	(43.9, 81.0)
25+	13.2% (101/764)		101	1460	6.9		(5.7, 8.4)	44.9% (97/216)		97	299.3	32.4		(26.6, 39.6)
Black racial identity (inclusive of those who identify as Black and another racial or ethnic identity)														
Yes	25.0% (57/228)	<0.001	57	389.8	14.6	<0.0001	(11.3, 19.0)	47.2% (51/108)	0.79	51	143.5	35.5	0.8264	(27.0, 46.8)
No	8.7% (80/918)		80	1726	4.6		(3.7, 5.8)	48.9% (85/174)		85	221.8	38.3		(31.0, 47.4)
Hispanic/Latina (inclusive of all racial identities)														
Yes	18.0% (34/189)	0.006	34	332.5	10.2	0.0043	(7.3, 14.3)	44.6% (37/83)	0.427	37	115.5	32.0	0.531	(23.2, 44.2)
No	10.9% (105/961)		105	1786.5	5.9		(4.9, 7.1)	49.8% (101/203)		101	252.5	40.0		(32.9, 48.6)
Region														
Northeast	11.9% (60/504)	0.073	60	874.3	6.9	0.0467	(5.3, 8.8)	49.6% (60/121)	0.806	60	152.5	39.3	0.7638	(30.5, 50.7)
Midwest	6.5% (9/138)		9	289.5	3.1		(1.6, 6.0)	41.2% (7/17)		7	19.5	35.9		(17.1, 75.3)
South	13.7% (70/513)		70	963	7.3		(5.8, 9.2)	48.0% (71/148)		71	196	36.2		(28.7, 45.7)
Cohort mode														
Site‐based technology enhanced	17.3% (103/594)	<0.001	103	995.3	10.3	<0.0001	(8.5, 12.6)	47.3% (106/224)	0.55	106	298.3	35.5	0.7258	(29.4, 43.0)
Exclusively digital	6.4% (36/561)		36	1131.5	3.2		(2.3, 4.4)	51.6% (32/62)		32	69.8	45.9		(32.4, 64.9)
Enrolment year														
2018	19.8% (67/338)	<0.001	67	574.5	11.7	<0.0001	(9.2, 14.8)							
2019	8.8% (66/748)		66	1444	4.6		(3.6, 5.8)							
2020	8.7% (6/69)		6	108.3	5.5		(2.5, 12.3)							
Income														
Above the federal poverty level	11.5% (73/635)	0.162	73	1189	6.1	0.0854	(4.9, 7.7)	44.1% (60/136)	0.397	60	187.5	32.0	0.2891	(24.8, 41.2)
Below the federal poverty level	14.5% (50/344)		50	600	8.3		(6.3, 11.0)	52.6% (61/116)		61	139.5	43.7		(34.0, 56.2)
Unknown	9.1% (16/176)		16	337.8	4.7		(2.9, 7.7)	50.0% (17/34)		17	41.0	41.5		(25.8, 66.7)
Educational background														
High school diploma/GED or less	18.0% (55/305)	<0.001	55	525.8	10.5	<0.0001	(8.0, 13.6)	57.9% (62/107)	0.038	62	119.0	52.1	0.0109	(40.6, 66.8)
Some college or higher	9.6% (81/840)		81	1585	5.1		(4.1, 6.4)	42.3% (74/175)		74	242.8	30.5		(24.3, 38.3)
Unknown	30.0% (3/10)		3	16.3	18.5		(6.0, 57.2)	50.0% (2/4)		2	6.3	32.0		(8.0, 128.0)
Health insurance^a^														
Uninsured	13.3% (26/195)	0.004	26	387.8	6.7	0.0119	(4.6, 9.8)	50.0% (22/44)	0.694	22	57.8	38.1	0.9498	(25.1, 57.9)
Public insurance (e.g. Medicaid or Medicare)	15.0% (69/460)		69	869.3	7.9		(6.3, 10.1)	50.0% (76/152)		76	205.8	36.9		(29.5, 46.3)
Private insurance	8.2% (38/466)		38	836.3	4.5		(3.3, 6.2)	44.3% (35/79)		35	90.8	38.6		(27.7, 53.7)
Experienced homelessness^a^														
No	9.9% (88/893)	<0.001	88	1652.3	5.3	<0.0001	(4.3, 6.6)	46.0% (92/200)	0.245	92	267.5	34.4	0.18	(28.0, 42.2)
Yes	19.8% (51/258)		51	471.5	10.8		(8.2, 14.2)	53.5% (46/86)		46	100.5	45.8		(34.3, 61.1)
PrEP indicated over follow‐up														
No	1.8% (7/396)	<0.001	7	755	0.9	<0.0001	(0.4, 1.9)	57.1% (8/14)	0.495	8	15	53.3	0.3891	(26.7, 106.6)
Yes	17.4% (132/759)		132	1371.8	9.6		(8.1, 11.4)	47.8% (130/272)		130	353	36.8		(31.0, 43.7)
PrEP initiation year														
2018								42.1% (40/95)	0.002	40	163.8	24.4	0.0028	(17.9, 33.3)
2019								52.9% (65/123)		65	157.5	41.3		(32.4, 52.6)
2020								65.1% (28/43)		28	34.5	81.2		(56.0, 117.5)
2021								27.8% (5/18)		5	9.8	51.3		(21.3, 123.2)
PrEP experience at enrolment														
PrEP‐naive	9.7% (102/1054)	<0.001	102	1985.3	5.1	<0.0001	(4.2, 6.2)	53.9% (55/102)	0.301	55	91.5	60.1	0.0044	(46.1, 78.3)
Former PrEP users	35.4% (35/99)		35	137.3	25.5		(18.3, 35.5)	51.4% (18/35)		18	39.3	45.9		(28.9, 72.8)
Current PrEP users								44.2% (65/147)		65	235	27.7		(21.7, 35.3)

^a^ p = 0.05

### 3.3 Predictors of PrEP initiation and discontinuation

In multivariable time‐to‐event models (Table 3), those who identified as Black had a 54% increase in PrEP initiation compared to those who did not identify as Black, while discontinuation did not differ by race. Those who participated in the exclusively digital cohort mode had a 46% decrease in PrEP initiation compared to those who were site‐based. Those who experienced PrEP indication had a seven‐fold increase in PrEP initiation (compared to those who were not indicated). Finally, those who were former PrEP users at baseline had a nearly three‐fold increase in PrEP initiation (compared to those who were PrEP naive).

**Table 3:** Multivariable Cox regression models for PrEP initiation and discontinuation among transgender women in the United States enrolled in The LITE Cohort and under observation March 2018–July 2022

	PrEP initiation			PrEP discontinuation		
Factor	Adjusted hazard ratio	*p*‐value	95% confidence interval	Adjusted hazard ratio	*p*‐value	95% confidence interval
Youth (ref: age 25+)	0.82	0.338	0.55, 1.23	1.40	0.083	0.96, 2.06
Black race (inclusive of those who identify as Black and another racial or ethnic identity)	1.54	0.038	1.03, 2.31	0.72	0.117	0.48, 1.09
Latina ethnicity (inclusive of all racial identities)	0.99	0.975	0.64, 1.54	0.90	0.630	0.59, 1.38
Census region (ref: northeast)						
Midwest	1.03	0.937	0.46, 2.31	0.62	0.298	0.26, 1.52
South	0.91	0.646	0.62, 1.34	0.94	0.734	0.66, 1.34
Exclusively digital cohort mode (ref: site‐based)	0.54	0.033	0.30, 0.95			
Enrolment year (ref: 2018)						
2019	0.72	0.146	0.47, 1.11			
2020	0.93	0.879	0.38, 2.28			
PrEP initiation year (ref: 2018)						
2019				1.33	0.182	0.87, 2.03
2020				1.80	0.044	1.01, 3.21
2021				0.98	0.964	0.34, 2.76
Education (ref: high school/GED or less)						
Some college or higher	0.85	0.396	0.57, 1.25	0.55	0.003	0.38, 0.81
Unknown	3.37	0.049	1.01, 11.25	0.55	0.414	0.13, 2.33
Insurance (ref: uninsured)						
Public insurance (e.g. Medicaid or Medicare)	0.89	0.646	0.54, 1.46			
Private insurance	1.04	0.874	0.61, 1.78			
Experienced homelessness	1.14	0.492	0.78, 1.68			
PrEP indicated over follow‐up	6.87	<0.001	3.17, 14.91			
PrEP experience at enrolment (ref: PrEP Naive)						
Former PrEP user	2.72	<0.001	1.78, 4.14	0.79	0.397	0.45, 1.37
Current PrEP user				0.66	0.050	0.43, 1.00

In terms of PrEP discontinuation, those who initiated PrEP in 2020 had an 80% increase in discontinuation (compared to those who initiated PrEP in 2018). Those with some college education or higher had a 45% decrease in discontinuation (compared to those with a high school education or less) and those who were current PrEP users at enrolment had a 34% decrease in discontinuation compared to those who used PrEP for the first time while under observation. Although marginally significant (*p* = 0.083), youth (those aged 18–24) had a 40% increase in discontinuation compared to those aged 25 years and older.

### 3.4 HIV seroconversion

Among 15 seroconversions observed in the study, seven occurred in transgender women without a reported history of PrEP use. One participant declined to disclose PrEP experience at enrolment but confirmed she was not a current user at subsequent study visits. The remaining seven seroconversions were among transgender women who reported prior PrEP use. Six of the seven reported PrEP use at just one study visit. The remaining participant was a former PrEP user at enrolment and reinitiated PrEP on two occasions while under observation (i.e. engaged in episodic use—sometimes called “prep cycling”). The time between last PrEP use and date of seroconversion ranged from 5 months to 2.9 years (median: 10 months).

### 3.5 Typologies of PrEP discontinuation

**Figure 2:**
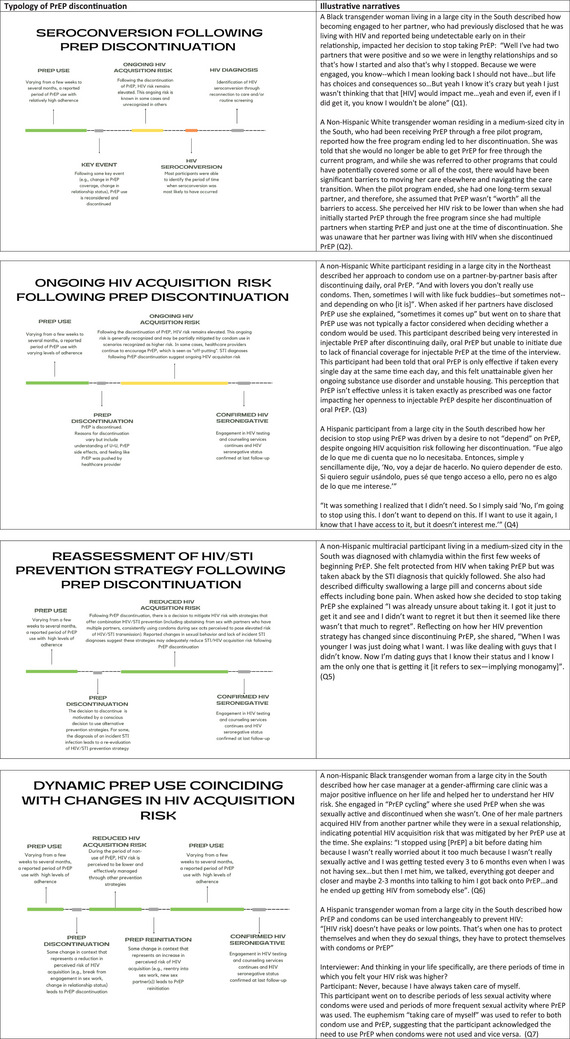
Typologies of PrEP discontinuation among transgender women in the United States enrolled in The LITE Cohort under observation March 2018–July 2022.

Integrating the qualitative and quantitative findings, four typologies of discontinuation emerged (Figure 2). The first was characterized by seroconversion following PrEP discontinuation. With this typology, there was a general awareness of HIV acquisition risk and demonstrated willingness to use PrEP, under certain circumstances. However, some key events (e.g. change in financial coverage for PrEP, relationship status, socio‐economic context) prompted a reassessment of the role and utility of PrEP, resulting in discontinuation (Q1 and Q2). HIV acquisition risk remained elevated (consistently or intermittingly), leading to HIV seroconversion several months to a few years after discontinuation.

Typology two was similarly characterized by a period of ongoing HIV acquisition risk following discontinuation but without seroconversion. Participants in this typology may have initiated PrEP after consistent encouragement from a healthcare provider, and the decision to initiate PrEP was characterized by some hesitation. While on PrEP, adherence varied, but generally PrEP use was brief (<6 months). Following discontinuation, several participants reported continued conversations about PrEP with healthcare providers, which one participant described as “off‐putting.” STI diagnoses and reports of condomless sex with multiple sex partners following discontinuation suggest ongoing HIV acquisition risk (Q3). Participants remained engaged in HIV testing and counselling through the study and/or outside organizations and were confirmed to be HIV seronegative at last follow‐up (Q4).

Typology three was characterized by a conscious decision to adjust one's strategy for reducing HIV/STI acquisition risk following discontinuation. This typology is defined by an initial period of high PrEP adherence and confidence in the HIV protection provided. For some, an Sexually transmitted infection (STI) diagnosis while on PrEP prompted the re‐evaluation of HIV/STI prevention strategy. The desire to prevent future STIs led to behavioural intention to use condoms consistently and/or abstain from sexual activities perceived to be higher risk (e.g. condomless anal or vaginal sex with partners who have more than one partner). With a commitment to incorporating these STI prevention practices, there was a recognition that the additional protection provided by continuing PrEP would be marginal. PrEP was, therefore, discontinued, and alternative strategies to reduce the risk of HIV/STI were implemented (Q5). Participants in this discontinuation typology remained engaged in HIV/STI testing and counselling services and were confirmed to be seronegative at last follow‐up.

The final typology of PrEP discontinuation was characterized by episodic PrEP use, or “PrEP cycling,” which, in this case, generally aligned with HIV acquisition risk (Q6 and Q7). Following a period of high PrEP adherence, change in socio‐behavioural context (e.g. disengagement from sex work, change in housing stability or period of abstinence from sex) prompted PrEP discontinuation. However, after time, another change (e.g. re‐engagement in sex work or engagement with new sex partners) prompted PrEP re‐initiation. Confirmation of seronegative status suggests that these discontinuations may be part of a prevention‐effective adherence trajectory.

## 4 DISCUSSION

We observed low PrEP initiation rates and high discontinuation rates among transgender women in the eastern and southern United States. Seventy‐eight percent remained unengaged in PrEP services over follow‐up, despite counselling and referrals. Transgender women who used PrEP while under observation followed heterogeneous patterns of PrEP use, including one‐fifth who engaged in “PrEP cycling,” characterized by at least one re‐initiation after discontinuation. Given how common PrEP cycling is in this population, clinical supports and programmes should acknowledge this approach and provide tailored support for trans women intending to implement this strategy.

Black and Latina transgender women, who shoulder an HIV burden several‐fold higher than non‐Hispanic White transgender women in the United States [2, 27], had higher rates of initiation compared to those who identified as non‐Hispanic White. However, rates of discontinuation did not differ by race or ethnicity, and 93% of HIV seroconversions in the sample occurred among Black and Latina participants, pointing to the ongoing need to address ethnoracial inequities in PrEP access and engagement among transgender women [4]. It is possible that community‐based programmes and campaigns partially explain the higher PrEP initiation rates among Black trans women; complementary campaigns and programmes are likely needed to support effective adherence and continuation.

The finding that participants in the exclusively digital cohort mode were less likely to initiate PrEP compared to those in the site‐based mode could reflect less PrEP access, which may be due to a larger proportion residing in rural areas. Previous analyses of data from this cohort have found lower healthcare access and less connection to health facilities among participants in the digital mode [4]. Transgender women with a college education or higher were less likely to discontinue PrEP. This finding corroborates previous studies that have established an association between higher socio‐economic status and PrEP persistence [11, 28]. Discontinuation rates trended higher among youth, which may be attributed to more dynamic PrEP indication [17]. We also found that those who initiated PrEP in 2020 had higher rates of discontinuation compared to those who initiated in 2018. One possible explanation is that transgender women who initiated PrEP in the first year of the COVID‐19 pandemic may have received less support due to widespread changes and disruptions throughout the healthcare system during the pandemic.

Discontinuation typologies highlight the heterogeneity of PrEP experiences and the dynamic nature of PrEP indication and use in this population. The first two typologies demonstrated ongoing HIV acquisition risk following PrEP discontinuation. For some transgender women, alternative prevention strategies following PrEP discontinuation were used but failed to meet HIV prevention needs. Consequently, seroconversions following discontinuation did occur. Longer‐acting PrEP modalities may better meet the needs of these transgender women, who were engaged in healthcare and found PrEP acceptable. This typology also raises the importance of not only reducing barriers to PrEP initiation, but also sustaining adherence support throughout PrEP use. The introduction of new barriers while already using PrEP (e.g. the financial and logistical burden posed when an existing PrEP programme ends) can directly lead to discontinuations and, subsequently, seroconversions.

As observed with the third discontinuation typology, some transgender women effectively met their HIV and STI prevention goals through non‐PrEP strategies following PrEP discontinuation (e.g. condom use, regular HIV/STI testing, changes in sexual partnerships and/or types of sex engaged in). There is unmet need for combination HIV/STI prevention products, which better align with the values and preferences of transgender women who discontinued PrEP for this reason. This typology is characterized by the adaptation of prevention strategies and shows the critical thought and reflection that goes into decision‐making around sexual health, complementing previously published findings on multiple HIV prevention strategies implemented by transgender women [29]. Thus, conversations about PrEP discontinuation with healthcare providers should utilize shared decision‐making approaches so that transgender women are supported in achieving their HIV/STI prevention goals irrespective of PrEP use.

Finally, as the fourth typology illustrates, some PrEP discontinuations are more accurately classified as pauses or hiatuses. Many transgender women reinitiated PrEP following a perceived change in HIV risk, had high PrEP adherence throughout periods of perceived HIV acquisition risk and discontinued again when appropriate. This group may benefit from forthcoming PrEP modalities (such as implants and infrequent injections) as they likely would be just as effective but would require less conscious decision‐making about HIV risk at a given time.

It is important to note that PrEP discontinuation typologies do not represent the full range of experiences and possibilities. Further, because some people may have multiple discontinuations, an individual could have multiple discontinuations representative of multiple typologies. Nevertheless, these typologies, which resulted from our inductive analytic approach and integration of mixed methods data, provide descriptions of common discontinuation narratives. They are, therefore, useful for categorizing discontinuation experiences and for informing tailored interventions to better support transgender women with ongoing HIV acquisition risk.

This study is limited by the biases inherent in self‐reported data. However, a recent evaluation assessing the validity of PrEP self‐report among clinic patients in NYC found that self‐reported PrEP adherence is highly accurate [30]. Recruitment for the site‐based cohort mode leveraged partnerships with gender‐affirming healthcare clinics, community‐based organizations and social service providers that serve transgender communities. Therefore, it is possible that this sample is more engaged in health and social services compared to the general population of transgender women, which may have led to an overestimation of PrEP engagement. It is also possible that barriers and circumstances surrounding discontinuation were more salient in the narratives of those who had seroconverted given the additional reflection on the health impact of the discontinuation. Future research to more fully expand upon the nuanced experiences of those who remain at elevated risk of HIV acquisition after PrEP discontinuation is needed. Finally, as qualitative data were limited to discontinuation experiences, there remains a need for a conceptual framework to integrate the multilevel factors impacting PrEP engagement more broadly among transgender women.

Despite these limitations, the integration of longitudinal quantitative and qualitative data from the only multi‐site cohort study of transgender women at risk for HIV in the United States provides important insight on PrEP initiation and discontinuation. The discontinuation typologies enhance our understanding of discontinuation experiences among transgender women so that HIV prevention interventions can better support transgender women to engage in prevention‐effective adherence practices.

## 5 CONCLUSIONS

Interventions to support and optimize transgender women's use of PrEP, alongside equitable scale‐up of long‐acting methods, are needed to address the disproportionate burden of HIV among transgender women. Supporting transgender women to meet their HIV prevention goals and addressing socio‐structural barriers to achieving these goals should be a central component of PrEP programming and interventions for transgender women.

## COMPETING INTERESTS

The authors declare no competing interests.

## AUTHORS’ CONTRIBUTIONS

ALW and SLR conceived of the LITE cohort, secured funding and led the overall project. EEC designed the analysis plan, wrote all statistical analysis code, produced all tables and figures, and wrote the first draft. HTS, ALW, SLR, MS, RA‐AR, KNA, SWB, TCP, CB and KHM provided substantive input on the analytic approach and interpretation of findings. EEC, ALW and SLR had full access to all data in the study, have verified the underlying data and accept responsibility to submit for publication. EEC, ALW and SLR obtained ethics approvals. ALW, AR, CMC, JSS, JSH, AER, AJW, SLR and KHM led study implementation at each site. All authors reviewed and approved the final manuscript.

6

## FUNDING

EEC was supported by a predoctoral fellowship from the National Institute of Mental Health (F31MH124582). The LITE study was jointly supported by the National Institute of Allergy and Infectious Diseases, the National Institute of Mental Health and the National Institute of Child Health and Human Development of the National Institutes of Health under Award Number UG3/UH3AI133669 (ALW and SLR). Research reported in this publication was also supported by HIV/AIDS, Hepatitis, STD and TB Administration (HAHSTA), Washington, DC, Department of Health. The LITE study is also appreciative of support from the CFAR at partner institutions, including JHU (P30AI094189), Emory University (P30AI050409), Harvard University (P30AI060354), DC CFAR (P30AI117970) and the University of Miami (P30AI073961).

## DISCLAIMER

The content is solely the responsibility of the authors and does not necessarily represent the official views of the National Institutes of Health or HAHSTA.

## Supporting information

Supporting Information

## Data Availability

De‐identified individual data, a data dictionary and code will be made available upon reasonable request after approval of a proposal and signing of a data use agreement. There is a formal process for external users to request access to LITE data, which involves review and approval by Principal Investigators from each study site as well as the Community Advisory Board; further details and forms can be obtained by emailing Dr. Andrea Wirtz (awirtz1@jhu.edu).

## References

[1] Baral SD , Poteat T , Stromdahl S , Wirtz AL , Guadamuz TE , Beyrer C . Worldwide burden of HIV in transgender women: a systematic review and meta‐analysis. Lancet Infect Dis. 2013;13(3):214–222. 23260128 10.1016/S1473-3099(12)70315-8

[2] Becasen JS , Denard CL , Mullins MM , Higa DH , Sipe TA . Estimating the prevalence of HIV and sexual behaviors among the US transgender population: a systematic review and meta‐analysis, 2006–2017. Am J Public Health. 2019;109(1):e1–e8. 10.2105/AJPH.2018.304727 PMC6301428 30496000

[3] Poteat T , Wirtz A , Malik M , Cooney E , Cannon C , Hardy WD , et al. A gap between willingness and uptake: findings from mixed methods research on HIV prevention among Black and Latina transgender women. J Acquir Immune Defic Syndr. 2019;82(2):131–140. 31180995 10.1097/QAI.0000000000002112 PMC7807529

[4] Wirtz AL , Humes E , Althoff KN , Poteat TP , Radix AE , Mayer KH et al. HIV incidence and mortality in a multi‐site cohort study of transgender women in the eastern and southern United States. Lancet HIV. 2023;10(5):e308–e319. 36868260 10.1016/S2352-3018(23)00008-5 PMC10164681

[5] Poteat T , Malik M , Scheim A , Elliott A . HIV prevention among transgender populations: knowledge gaps and evidence for action. Curr HIV/AIDS Rep. 2017;14(4):141–152. 28752285 10.1007/s11904-017-0360-1 PMC5896563

[6] Cahill SR , Keatley J , Wade Taylor S , Sevelius J , Elsesser SA , Geffen SR , et al. “Some of us, we don't know where we're going to be tomorrow.” Contextual factors affecting PrEP use and adherence among a diverse sample of transgender women in San Francisco. AIDS Care. 2020;32(5):585–593. 31482726 10.1080/09540121.2019.1659912 PMC12995398

[7] Baldwin A , Light B , Allison WE . Pre‐exposure prophylaxis (PrEP) for HIV infection in cisgender and transgender women in the US: a narrative review of the literature. Arch Sex Behav. 2021;50(4):1713–1728. 34075504 10.1007/s10508-020-01903-8 PMC8213571

[8] Malone J . Perceived HIV acquisition risk and low uptake of PrEP among a cohort of transgender women with PrEP indication in the eastern and southern United States. J Acquir Immune Defic Syndr. 2021;88(1):10–18. 34397742 10.1097/QAI.0000000000002726 PMC8371736

[9] Aldredge A , Roth G , Vaidya A , Paula Duarte A , Kundu S , Zheng Z , et al. Preexposure prophylaxis care continuum among transgender women at a patient‐centered preexposure prophylaxis program in Atlanta, Georgia. AIDS. 2021;35(3):524–526. 33507011 10.1097/QAD.0000000000002788

[10] Storholm ED , Ogunbajo A , Nacht CL , Opalo C , Horvath KJ , Lyman P , et al. Facilitators of PrEP persistence among Black and Latinx transgender women in a PrEP Demonstration Project in Southern California. Behav Med. 2022;22(4):1–12. 10.1080/08964289.2022.2105794 PMC9943802 35993278

[11] Konda KA , Torres TS , Marino G , Ramos A , Moreira RI , Leite IC , et al. Factors associated with long‐term HIV pre‐exposure prophylaxis engagement and adherence among transgender women in Brazil, Mexico and Peru: results from the ImPrEP study. J Int AIDS Soc. 2022;25 **Suppl 5**(Suppl 5):e25974. 36225148 10.1002/jia2.25974 PMC9557020

[12] Haberer JE , Bangsberg DR , Baeten JM , Curran K , Koechlin F , Amico KR , et al. Defining success with HIV pre‐exposure prophylaxis: a prevention‐effective adherence paradigm. AIDS. 2015;29(11):1277–1285. 26103095 10.1097/QAD.0000000000000647 PMC4480436

[13] Haberer JE . Current concepts for PrEP adherence in the PrEP revolution: from clinical trials to routine practice. Curr Opin HIV AIDS. 2016;11(1):10–17. 26633638 10.1097/COH.0000000000000220 PMC4801217

[14] Roberts DA , Bridenbecker D , Haberer JE , Barnabas RV , Akullian A . The impact of prevention‐effective PrEP use on HIV incidence: a mathematical modelling study. J Int AIDS Soc. 2022;25(11):e26034. 36385504 10.1002/jia2.26034 PMC9670193

[15] Spinelli MA , Laborde N , Kinley P , Whitacre R , Scott HM , Walker N , et al. Missed opportunities to prevent HIV infections among pre‐exposure prophylaxis users: a population‐based mixed methods study, San Francisco, United States. J Int AIDS Soc. 2020;23(4):e25472. 32294338 10.1002/jia2.25472 PMC7159249

[16] Velloza J , Donnell D , Hosek S , Anderson PL , Chirenje ZM , Mgodi N , et al. Alignment of PrEP adherence with periods of HIV risk among adolescent girls and young women in South Africa and Zimbabwe: a secondary analysis of the HPTN 082 randomised controlled trial. Lancet HIV. 2022;9(10):e680–e689. 36087612 10.1016/S2352-3018(22)00195-3 PMC9530001

[17] Cooney EE , Reisner SL , Saleem HT , Althoff KN , Beckham SW , Radix A , et al. Prevention‐effective adherence trajectories among transgender women indicated for PrEP in the United States: a prospective cohort study. Ann Epidemiol. 2022;70:23–31. 35398255 10.1016/j.annepidem.2022.03.016 PMC9167788

[18] Wirtz AL , Poteat T , Radix A , Althoff KN , Cannon CM , Wawrzyniak AJ , et al. American Cohort to Study HIV Acquisition Among Transgender Women in High‐Risk Areas (The LITE Study): protocol for a multisite prospective cohort study in the eastern and southern United States. JMIR Res Protoc. 2019;8(10):e14704. 31584005 10.2196/14704 PMC6802485

[19] Wirtz AL , Cooney EE , Stevenson M , Radix A , Poteat T , Wawrzyniak AJ , et al. Digital epidemiologic research on multilevel risks for HIV acquisition and other health outcomes among transgender women in eastern and southern United States: protocol for an online cohort. JMIR Res Protoc. 2021;10(4):e29152. 33900202 10.2196/29152 PMC8111508

[20] Akinola M , Wirtz AL , Chaudhry A , Cooney E , Reisner SL ; American Cohort to Study HIVAATW . Perceived acceptability and feasibility of HIV self‐testing and app‐based data collection for HIV prevention research with transgender women in the United States. AIDS Care. 2021;33(8):1079–1087. 33487032 10.1080/09540121.2021.1874269 PMC8298585

[21] Reisner SL , Chaudhry A , Cooney E , Garrison‐Desany H , Juarez‐Chavez E , Wirtz AL . ‘It all dials back to safety’: a qualitative study of social and economic vulnerabilities among transgender women participating in HIV research in the USA. BMJ Open. 2020;10(1):e029852. 10.1136/bmjopen-2019-029852 PMC7044992 31959600

[22] Wirtz AL , Cooney EE , Chaudhry A , Reisner SL ; American Cohort To Study HIVAATW . Computer‐mediated communication to facilitate synchronous online focus group discussions: feasibility study for qualitative HIV research among transgender women across the United States. J Med Internet Res. 2019;21(3):e12569. 30924782 10.2196/12569 PMC6460306

[23] Cooney EE . HIV pre‐exposure prophylaxis engagement trajectories among transgender women in the United States. Johns Hopkins University; 2023.

[24] Atkinson R . The life story interview. Sage; 1998.

[25] Nelson IA . From quantitative to qualitative: adapting the life history calendar method. Field Methods. 2010;22(4):413–428.

[26] Fetters MD , Curry LA , Creswell JW . Achieving integration in mixed methods designs‐principles and practices. Health Serv Res. 2013;48(6 Pt 2):2134–2156. 24279835 10.1111/1475-6773.12117 PMC4097839

[27] Centers for Disease Control and Prevention . HIV infection, risk, prevention, and testing behaviors among transgender women—National HIV Behavioral Surveillance, 7 U.S. cities, 2019–20. 2021.

[28] Grinsztejn B , Hoagland B , Moreira RI , Kallas EG , Madruga JV , Goulart S , et al. Retention, engagement, and adherence to pre‐exposure prophylaxis for men who have sex with men and transgender women in PrEP Brasil: 48 week results of a demonstration study. Lancet HIV. 2018;5(3):e136–e145. 29467098 10.1016/S2352-3018(18)30008-0

[29] Aguayo‐Romero RA , Cannon CM , Wirtz AL , Cooney EE , Mayer KH , Reisner SL , et al. HIV awareness and prevention strategies among transgender women in the Eastern and Southern United States: findings from the LITE Study. J Int AIDS Soc. 2022;25 **Suppl 5**(Suppl 5):e25999. 36225140 10.1002/jia2.25999 PMC9557018

[30] Qasmieh S , Nash D , Gandhi M , Rozen E , Okochi H , Goldstein H , et al. Self‐reported use of HIV preexposure prophylaxis is highly accurate among sexual health clinic patients in New York City. Sex Transm Dis. 2022;49(11):790–793. 35312670 10.1097/OLQ.0000000000001622 PMC9463403

