# Supplementary material for: PrEP initiation and discontinuation among transgender women in the United States: a longitudinal, mixed methods cohort study
Source: J Int AIDS Soc. 2023 Dec 20;26(12):e26199. doi: 10.1002/jia2.26199 (PMC10733152; doi:10.1002/jia2.26199)
Supplement: Supplementary file 1 — Supporting Information [file JIA2-26-e26199-s001.docx]

**SUPPLEMENTARY MATERIALS**

**PrEP discontinuation among transgender women in the United States: a longitudinal, mixed methods cohort study**

**Authors:** Erin E. Cooney, MSPH^1^, Haneefa T. Saleem, PhD^1^, Meg Stevenson, MSPH^2^, Rodrigo A. Aguayo-Romero, PhD^3,4,5^, Keri N. Althoff, PhD^2^, Tonia C. Poteat, PhD^6^, S. Wilson Beckham, PhD^7^, Dee Adams, MSPH^2^, Asa E. Radix, MD^8^, Andrew J. Wawrzyniak, PhD^9^, Christopher M. Cannon, MPH^10^, Jason S. Schneider, MD^11^, J. Sonya Haw, MD^12^, Allan E. Rodriguez, MD^13^, Prof Kenneth H. Mayer^4,5^, MD, Prof Chris Beyrer, MD^14^, Sari L. Reisner, PhD^3,4,5,15^*, Andrea L. Wirtz, PhD^1,2^* on behalf of American Cohort to Study HIV Acquisition Among Transgender Women (LITE) Study Group

^1^Johns Hopkins Bloomberg School of Public Health, Department of International Health, Baltimore, United States

^2^Johns Hopkins Bloomberg School of Public Health, Department of Epidemiology, Baltimore, United States

^3^Brigham and Women’s Hospital, Division of Endocrinology, Diabetes, and Hypertension, Boston, United States

^4^Harvard Medical School, Department of Medicine, Boston, United States

^5^Fenway Health, The Fenway Institute, Boston, United States

^6^University of North Carolina, Department of Social Medicine, Chapel Hill, United States

^7^Johns Hopkins Bloomberg School of Public Health, Department of Health, Behavior and Society, Baltimore, United States

^8^Callen-Lorde Community Health Center, New York, United States

^9^University of Miami Miller School of Medicine, Department of Psychiatry and Behavioral Sciences, Miami, United States

^10^Whitman-Walker Institute, Washington, D.C., United States

^11^Emory University School of Medicine, Department of Medicine, Atlanta, United States

^12^Emory University School of Medicine, Division of Endocrinology, Metabolism and Lipids, Atlanta, United States

^13^University of Miami Miller School of Medicine, Division of Infectious Diseases, Department of Medicine, Miami, United States

^14^Duke University, Global Health Institute, Durham, United States

^15^Harvard TH Chan School of Public Health, Department of Epidemiology, Boston, United States

*Co-senior authorship

**Corresponding Author:**

Dr. Erin Cooney

Department of International Health

Bloomberg School of Public Health

Johns Hopkins University

Baltimore, MD, 21205, USA

Ecooney2@jhmi.edu

+1 401-595-3436

**Supplementary Panel 1: Operational definitions of PrEP engagement and indication**

**PrEP experience at enrollment**

- ***PrEP-naïve***: responded “no” to “Have you **ever** taken PrEP (pre-exposure prophylaxis) for the prevention of HIV infection?”
- ***Former PrEP user***: responded “yes” to “Have you **ever** taken PrEP (pre-exposure prophylaxis) for the prevention of HIV infection?” and “no” to “Are you **currently** taking PrEP (pre-exposure prophylaxis) for the prevention of HIV infection (in the last 30 days)?”
- ***Current PrEP user***: responded “yes” to “Are you **currently** taking PrEP (pre-exposure prophylaxis) for the prevention of HIV infection (in the last 30 days)?

**Current PrEP use over follow-up**

- Responded “yes” to the question “Are you **currently** taking PrEP (pre-exposure prophylaxis) for the prevention of HIV infection (in the last 30 days)?” at a given timepoint
- Response options changed to “yes, oral PrEP” and “yes, injectable PrEP” following the FDA approval of injectable cabotegravir for HIV prevention in December 2021. However, only n=3 reported injectable PrEP use and all were also oral PrEP users. Thus, except where noted, current PrEP use refers to use of daily, oral PrEP.

**PrEP initiation**

- Self-reported current PrEP use for the first time (among those who were PrEP-naïve at enrollment) or following a period of non-use (among former users at enrollment)
- Initiations are thus inclusive of re-starts among former PrEP users at enrollment
- Among participants who engaged in episodic PrEP use or “PrEP cycling” (and therefore had >1 initiation while under observation), the visit where PrEP use was first reported was considered the time of PrEP initiation

**PrEP discontinuation**

- Self-reported PrEP non-use following a period of current PrEP use
- Inclusive of those who initiated PrEP while under observation and those who were current users at enrollment
- Among participants who engaged in PrEP cycling (and therefore had >1 discontinuation while under observation), the final discontinuation was considered the time of PrEP discontinuation

**PrEP indication**

- Composite variable based on CDC PrEP prescribing guidelines, which have been adapted specifically for transgender women^8^
- PrEP indicated if:
  - Biologically confirmed HIV-negative serostatus
  - Had a sex partner with flesh penis within the past 6 months
  - Not in a monogamous sexual partnership with an HIV-negative partner
  - AND reported at least one of the following recent indicators (i.e., within past 3 months):
    - (a) condomless anal sex
    - (b) STI diagnosis
    - (c) sex work
    - (d) use of postexposure prophylaxis
    - (e) condomless anal or vaginal sex with a partner living with HIV or of unknown HIV status
    - (f) needle sharing

**Supplementary Figure 1: STROBE Diagram for The LITE Study**


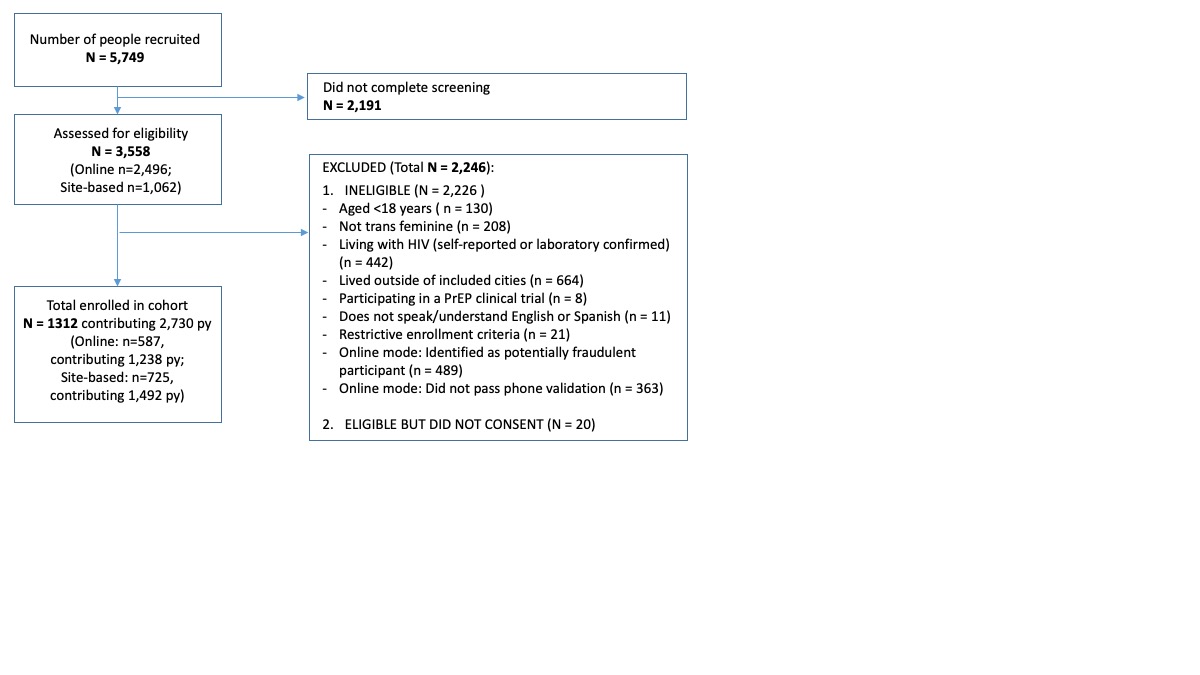


| **Supplementary Table 1. Sociodemographic characteristics of qualitative interviewees (n=18)** | |
| --- | --- |
| Factor |  |
| N | 18 |
| Age, median (range) | 30 (18, 50) |
| Age category |  |
| Youth (18-24 years) | 4 (22%) |
| Age 25+ years | 14 (78%) |
| Ethnoracial identity |  |
| Non-Hispanic White | 2 (11%) |
| Non-Hispanic Black | 8 (44%) |
| Hispanic White | 2 (11%) |
| Hispanic Black | 1 (6%) |
| Non-Hispanic and more than one race or other race | 1 (6%) |
| Hispanic and more than one race or other race | 4 (22%) |
| Black racial identity (inclusive of those who identify as Black and another racial or ethnic identity) |  |
| No | 8 (44%) |
| Yes | 10 (56%) |
| Hispanic/Latina (inclusive of all racial identities) |  |
| No | 11 (61%) |
| Yes | 7 (39%) |
| Census region |  |
| Northeast | 2 (11%) |
| Midwest | 1 (6%) |
| South | 15 (83%) |
| Cohort mode |  |
| Site-based technology enhanced | 16 (89%) |
| Exclusively digital | 2 (11%) |
| Year of study enrollment |  |
| 2018 | 12 (67%) |
| 2019 | 6 (33%) |
| Income |  |
| Above the federal poverty level | 6 (33%) |
| Below the federal poverty level | 7 (39%) |
| Unknown | 5 (28%) |
| Educational background |  |
| High school diploma/GED or less | 12 (67%) |
| Some college or higher | 6 (33%) |
| Health Insurance^ |  |
| Uninsured | 4 (24%) |
| Public insurance (e.g., Medicaid or Medicare) | 8 (47%) |
| Private insurance | 5 (29%) |
| Experienced homelessness^ |  |
| No | 13 (72%) |
| Yes | 5 (28%) |
| PrEP indicated at enrollment |  |
| No | 4 (22%) |
| Yes | 14 (78%) |
| PrEP indicated over follow-up |  |
| Yes | 18 (100%) |
| PrEP experience at enrollment |  |
| PrEP Naive | 10 (56%) |
| Former PrEP User | 3 (17%) |
| Current PrEP User | 5 (28%) |
| PrEP use and HIV serostatus over follow-up in LITE cohort* |  |
| Initiated PrEP during follow-up | 9 (50%) |
| Discontinued PrEP during follow-up | 11 (61%) |
| Seroconverted during follow-up | 3 (17%) |
| ^Participants were considered uninsured and to have experienced homelessness if uninsured status and recent homelessness were reported at any point over follow-up, respectively. The remaining characteristics were reported based on response at enrollment (except where noted).  *Categories are not mutually exclusive. | |
